# Tim-3-mediated signaling in NK cells may be modulated by increased Galectin-9 expression in HIV-1 infection

**DOI:** 10.1186/1742-4690-9-S2-P173

**Published:** 2012-09-13

**Authors:** S Jost, UY Moreno-Nieves, WF Garcia Beltran, K Rands, J Reardon, I Toth, A Piechocka-Trocha, BD Walker, M Altfeld, MM Addo

**Affiliations:** 1 Ragon Institute of MGH, MIT and Harvard, Boston, MA, USA

## Background

Natural Killer (NK) cells constitutively express high levels of Tim-3, an immunoregulatory molecule recently proposed to be a marker for mature and fully functional NK cells (Ndhlovu et al., 2012). Cytokines can induce Tim-3 expression on NK cells, and IFN-gamma production by Tim-3+ NK cells can be enhanced by exposure to Galectin-9 (Gleason et al. 2012), yet their ability to kill target cells is lost upon Tim-3 cross-linking. Moreover, up-regulation of Tim-3 on NK cells has been associated with reduced anti-viral properties in chronic hepatitis B infection (Ju et al., 2010). However, the impact of HIV-1 infection on Tim-3 expression on NK cells and on Tim-3-mediated NK cell function has not been studied yet.

## Methods

Flow cytometry was used to analyze Tim-3 and intracellular galectin-9 expression in subjects with acute and chronic HIV-1 infection, in HIV-1 elite (VL <50 copies/ml) and viremic controllers (VL <2000 copies/ml), and in HIV-1 negative subjects. Plasma levels of Galectin-9 were quantified by ELISA.

## Results

HIV-1 infection was associated with reduced expression of Tim-3 on NK cells, as early as in acute infection, and could be normalized by HAART. Importantly, percentages of Tim-3+ CD56_dim_ NK cells correlated with CD4+ cell counts in untreated patients. Plasma concentrations of Galectin-9 were higher in HIV-1-infected individuals than in controls. Interestingly, Galectin-9 expression in immune cells was significantly elevated in acute infection, with monocytes and dendritic cells displaying the highest levels, which correlated with viral loads. In vitro, Galectin-9 triggered NK cell activation and Tim-3 down-regulation on NK cells.

## Conclusion

Further investigations are warranted to determine whether increased Galectin-9 production alters Tim-3 function and contributes to NK cell impaired activity in chronic HIV-1 infection. Defining the role of NK cell receptors in the control of HIV-1 will offer novel therapeutic targets to manipulate and improve future HIV-1 vaccine strategies.

